# Accurate Bond Lengths to Hydrogen Atoms from Single‐Crystal X‐ray Diffraction by Including Estimated Hydrogen ADPs and Comparison to Neutron and QM/MM Benchmarks

**DOI:** 10.1002/chem.201604705

**Published:** 2017-03-15

**Authors:** Birger Dittrich, Jens Lübben, Stefan Mebs, Armin Wagner, Peter Luger, Ralf Flaig

**Affiliations:** ^1^ Heinrich-Heine Universität DüsseldorfInstitut für Anorganische Chemie und Strukturchemie, Material- und Strukturforschung, Gebäude: 26.42Universitätsstraße 140225DüsseldorfGermany; ^2^ Institut für Chemie und Biochemie–Anorganische Chemie derFreien Universität Berlin14195BerlinGermany; ^3^ Diamond Light SourceHarwell Science and Innovation CampusDidcotOX11 0DEUK

**Keywords:** amino acids, density functional theory, neutron diffraction, structure elucidation, X-ray diffraction

## Abstract

Amino acid structures are an ideal test set for method‐development studies in crystallography. High‐resolution X‐ray diffraction data for eight previously studied genetically encoding amino acids are provided, complemented by a non‐standard amino acid. Structures were re‐investigated to study a widely applicable treatment that permits accurate X−H bond lengths to hydrogen atoms to be obtained: this treatment combines refinement of positional hydrogen‐atom parameters with aspherical scattering factors with constrained “TLS+INV” estimated hydrogen anisotropic displacement parameters (H‐ADPs). Tabulated invariom scattering factors allow rapid modeling without further computations, and unconstrained Hirshfeld atom refinement provides a computationally demanding alternative when database entries are missing. Both should incorporate estimated H‐ADPs, as free refinement frequently leads to over‐parameterization and non‐positive definite H‐ADPs irrespective of the aspherical scattering model used. Using estimated H‐ADPs, both methods yield accurate and precise X−H distances in best quantitative agreement with neutron diffraction data (available for five of the test‐set molecules). This work thus solves the last remaining problem to obtain such results more frequently. Density functional theoretical QM/MM computations are able to play the role of an alternative benchmark to neutron diffraction.

## Introduction

Single‐crystal X‐ray structure analysis accurately and rapidly provides solid‐state connectivity, bond lengths, and angles. However, one weakness of the method remained. Bond lengths to hydrogen atoms used to be determined to be approximately 10 % shorter compared to those from neutron diffraction.^1^, ^2^, ^3^ This still holds for most analyses today and is due to the spherically symmetric atomic scattering factors of the commonly used independent atom model (IAM). Accurate bond lengths to hydrogen atoms can be obtained experimentally by neutron diffraction, possibly involving a correction for libration.^4^ Quantum chemical computations conveniently yield such distances for molecules in a hypothetical gas phase at a temperature of 0 K. When crystal‐field or packing effects are to be included in the quantum chemical description, a quantum mechanics/molecular mechanics (QM/MM) approach^5^, ^6^ is computationally most efficient. Full‐periodic computations are able to provide reference solid‐state results.^7^

Our first successful attempt to provide accurate experimental X−H bond lengths from single‐crystal X‐ray diffraction (XRD) made use of invariom refinement (INV).^8^ Invarioms are tabulated aspherical scattering factors^9^ that rely on the Hansen/Coppens multipole model.^10^, ^11^ Our study compared room‐temperature neutron data^12^ with the X‐ray results after invariom refinement, and it was shown that quantitative agreement of X‐ray and neutron diffraction within three standard uncertainties is also possible for X−H bond lengths. Hirshfeld atom refinement (HAR)^13^, ^14^ then allowed including the effect of neighboring molecules, which is mostly relevant for strong hydrogen bonds.^17^ HAR relies on a quantum‐chemical basis‐set model and gives best results in combination with density functional theory (DFT). Note that in HAR, initial IAM structures are subject to single‐point energy computations to give molecular electron density distributions that are Hirshfeld partitioned^15^ and Fourier transformed^16^ thereby providing atomic scattering factors tailor‐made for a molecule under study. These are then gradually fitted to give the best agreement to the diffraction data, and the procedure is repeated to convergence. HAR has recently been improved to automatically reach convergence^14^ and shown to give results in quantitative agreement to neutron diffraction; its strongest point remains to include crystal‐field effects in the model.

INV and HAR thus both overcome the limitations of conventional X‐ray diffraction with IAM scattering factors^8^, ^18^ and can now provide bond lengths to hydrogen atoms which are comparable to neutron diffraction.^14^ In 2005 only isotropic hydrogen displacement parameters were refined.^8^ In 2016, more accurate bond lengths to hydrogen atoms were obtained by refining H‐ADPs as well on a larger test‐set.^18^ However, it then turned out that H‐ADPs often become non‐positive definite. The current study solves this latter problem by investigating the use of estimated anisotropic displacement parameters (ADPs) for hydrogen atoms in comparison to free refinement of these sensitive parameters.

### Free refinement of H‐ADPs is hardly possible in general

Restraints (or constraints for riding hydrogen atoms) are not implemented in the program tonto,^19^ the only code that can currently perform HAR. Rather, linear dependencies in the least‐squares matrix are eliminated automatically.

**Figure 1:**
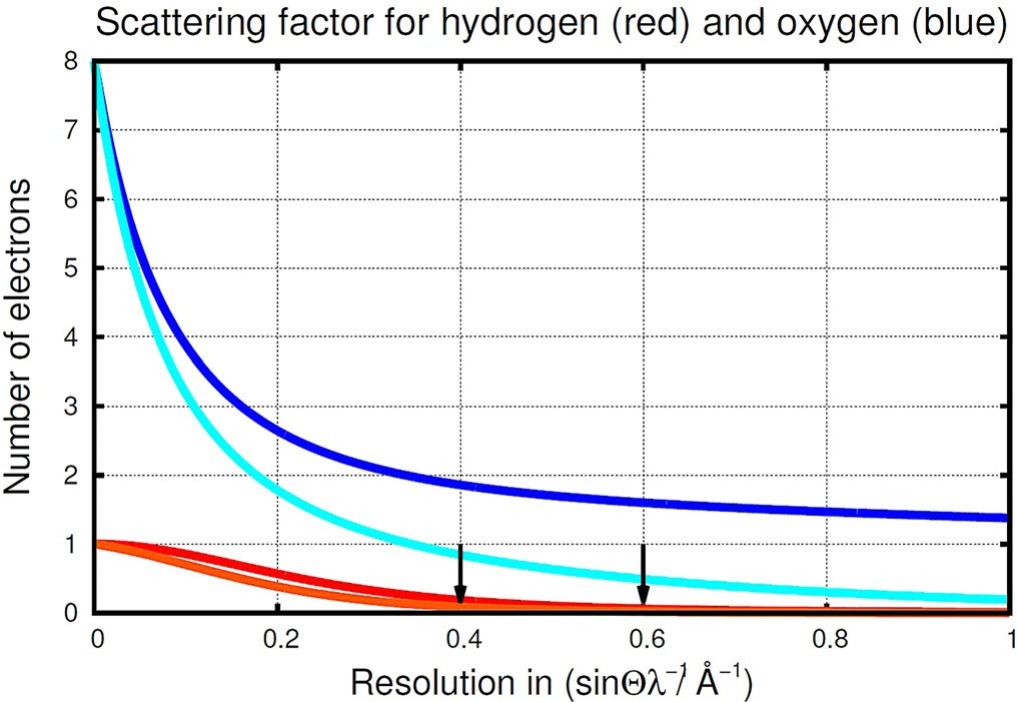
Static scattering for hydrogen (red) in comparison to oxygen (blue). The drop‐off with resolution is more pronounced for hydrogen as there are no core electrons. Thermal motion causes additional decay with resolution (light blue and orange lines); hydrogen scattering ceases to contribute to scattering at ca. 0.4 Å^−1^ with an isotropic temperature factor *T*=exp [−8π^2^⟨*u* ^2^⟩(sin^2^ *θ*/*λ* ^2^)] with *u* ^2^=0.025 Å^2^.

Earlier unpublished tests of free refinement of H‐ADPs with aspherical scattering factors from databases predicted by density functional theory (DFT) showed the same trends as HAR (that is, non‐positive H‐ADPs), and did therefore not convince the authors. Note that four scattering‐factor databases, or, more precisely, aspherical atomic density models parameterized in the multipole formalism, currently exist. On the experimental side the supramolecular‐synthon‐based fragments approach SBFA^20^ and the experimental library multipolar atom model ELMAM2^21^, ^22^ are both based on averaged experimental multipole parameters from high‐resolution diffraction experiments for chemical environments considered to be similar. Theoretical databases are the generalized invariom database GID,^23^ which is based on DFT geometry‐optimized model compounds using empirical rules of chemical similarity, and the University at Buffalo Databank UBDB2011,^24^, ^25^ which relies on DFT single‐point computations of experimental input structures with similarity deduced from statistical treatment. Our hope was that including crystal‐field effects^13^ would be the solution. However, we learned that simultaneous refinement of hydrogen positions and ADPs only succeeds with superior low‐order X‐ray diffraction data,^26^ preferably measured at very low temperatures. When X‐ray data up to (sin *θ*)/*λ*=0.6 Å^−1^, to which static hydrogen scattering is approximately limited, are imperfect, free refinement of positions and ADPs frequently leads to un‐physical results in terms of non‐positive definite (NPD) ADPs. Over‐parameterization becomes apparent: there are often not enough reflections that carry the relevant information to determine accurate experimental X−H bond lengths and H‐ADPs, and the latter might become NPD (see Figure 1). Note that the number of reflections *N* increases with resolution *N*=*V*4/3π(2(sin *θ*)/*λ*)^3^ depending on the cell volume *V*. While the number of reflections increases, the ratio of reflections carrying information on hydrogen scattering in a dataset decreases with increasing resolution. Therefore, higher resolution in a X‐ray experiment will not necessarily lead to better precision for hydrogen‐atom parameters, unless the data are measured at temperatures significantly below 100 K.

The situation is different for neutron diffraction, where the constant scattering length in combination with the comparatively large value of hydrogen/deuterium scattering puts hydrogen on par with other, heavier elements.

### Estimation of H‐ADPs

An alternative to free adjustment of hydrogen atom ADPs is to estimate these parameters. There are several ways to do so: the use of experimental infrared (IR) frequencies has been optimized^27^ following Hirshfeld's pioneering work.^28^ Appropriately scaled theoretical IR frequencies have also been used,^29^ which is possible when the conformation of the gas‐phase optimized molecule agrees with that found in the experimental solid state. When this is not the case the conformation can be maintained by ONIOM cluster computations.^30^ An approach used frequently is the shade server;^31^, ^32^ it relies on averaged internal displacements from neutron‐diffraction studies. Most recent work by the same authors can also rely on frequencies from lattice dynamics computations (adjusted to the X‐ray data) as a source of H‐ADPs.^33^ Correct temperature‐dependent behavior^34^ combined with automated segmented‐body TLS refinement is possible with the TLS+INV approach^35^ and the program APD‐toolkit, and this was the route followed herein.

### How to estimate H‐ADPs: the TLS+INV approach

Model compounds derived from an invariom name underlie the invariom approach. They contain the particular chemical environment of an atom of interest. Each atom in a structure is characterized by such a computer‐readable invariom name. The notation describes bonding to nearest and next‐nearest neighbors, and identifies an atom in a molecule. Model compounds are energy‐minimized (geometry‐optimized), followed by frequency computations as part of an automated procedure to get scattering factors. These infrared frequencies in turn can be used to compute internal atomic displacements, which, combined with a (segmented‐body) translation libration screw (TLS) fit, can provide good estimates of temperature‐dependent anisotropic motion of hydrogen atoms in a structure.^35^ Here it will be shown that it is recommendable to use such estimated hydrogen‐atom ADPs as constraints in aspherical‐atom (INV and HAR) crystallographic least‐squares refinements and to refine their positional parameters only, and to obtain even more accurate bond lengths to hydrogen atoms in this manner.

## Experimental Data and Least‐Squares Refinement

### Molecules studied

**Figure 2:**
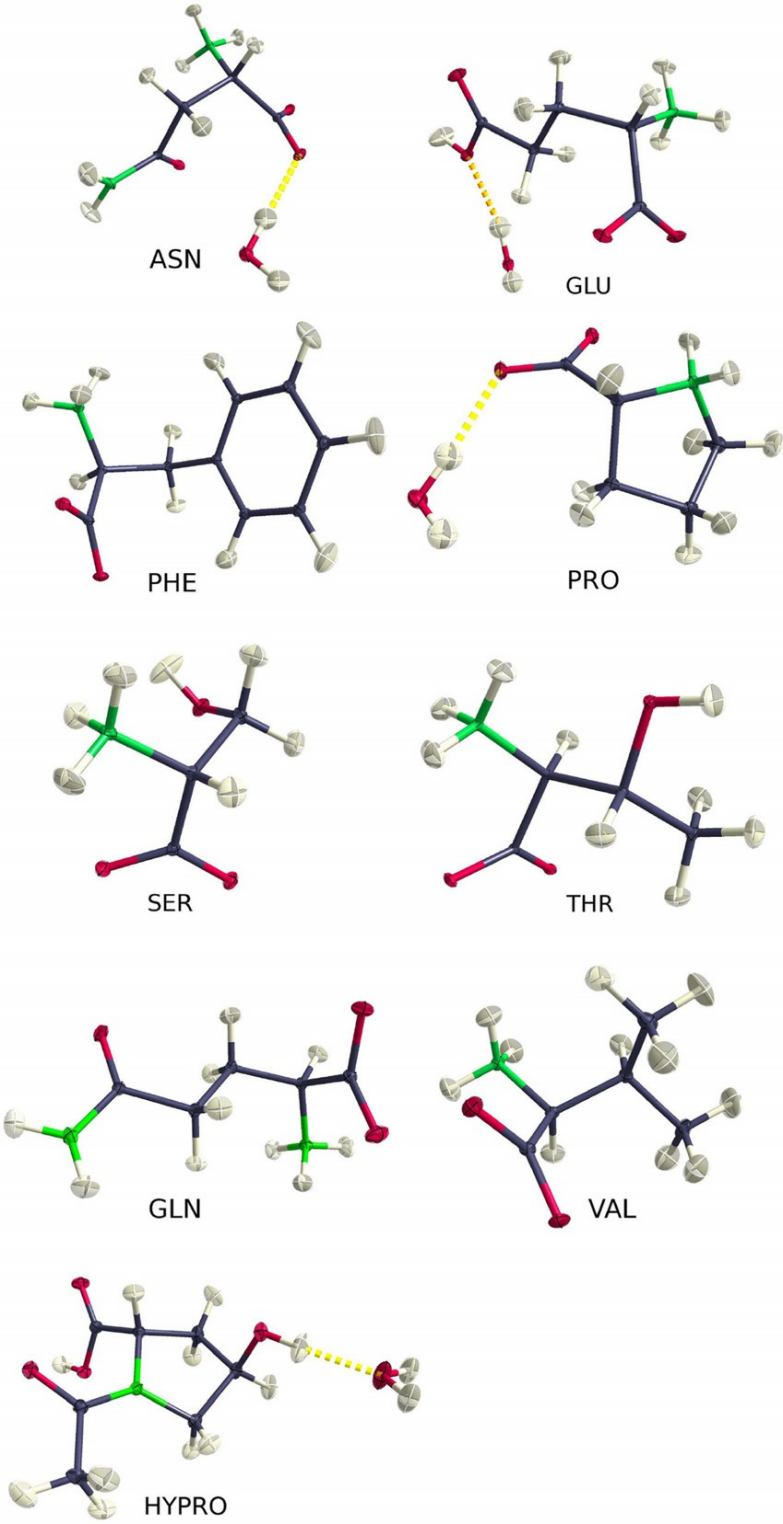
The ONIOM high‐layer (usually the asymmetric unit content except for l‐phenylalanine on the top right) of the nine structures investigated. Depictions show experimental ortep plots after invariom refinement, including TLS+INV estimated H‐ADPs as well as refined hydrogen positions. Depictions were generated with the program molecoolQt.^45^ ortep plots from HAR refinements with TLS+INV estimated H‐ADPs are visually indistinguishable.

The test set used in this work comprises of eight of the genetically encoded amino acids, relying on accurate previously analyzed structures. In these earlier studies, our diffraction data were not deposited, and they are now attached as Supporting Information together with refcodes of the respective CIF files. Structure factors can also be obtained from the CCDC via https://www.ccdc.cam.ac.uk/structures/. Moreover, the non‐standard amino acid *N*‐acetyl‐l‐4‐hydroxyproline monohydrate was added, since high‐resolution neutron as well as X‐ray diffraction data measured at low temperature are available^34^ (Figure 2, Table 1). A broad overview on structural work on this class of compounds of fundamental biological interest has recently been given.^36^ Data for d,l‐serine are those from an ultra‐high resolution synchrotron experiment as well as those from an earlier multi‐temperature study.^8^

**Table 1:** Selected crystallographic details of the nine structures studied.^[a]^

Structure	Space group	*Z*,*Z*′	*T* [K]	Source type	Reference
d,l‐asparagine H_2_O (ASN)	*P*2_1_2_1_2_1_	4,2	100	synchrotron	[37]
			RT	neutron	[38]
d,l‐glutamic Acid⋅H_2_O (GLU)	*Pbca*	8,2	100	synchrotron	[37]
l‐glutamine (GLN)	*P*2_1_2_1_2_1_	4,1	100	Mo Kα	[39]
			RT	neutron	[40]
l‐phenylalanine^[b]^ (PHE)	*P*2_1_	2,3	25	Mo Kα	[41]
d,l‐proline⋅H_2_O (PRO)	*Pbca*	8,2	100	synchrotron	[42]
d,l‐serine (SER)	*P*2_1_/*a*	4,1	100	synchrotron	[37]
			RT	neutron	[12]
l‐threonine (THR)	*P*2_1_2_1_2_1_	8,1	19	Ag Kα	[37]
			RT	neutron	[43]
d,l‐valine (VAL)	*P* $\bar{1}$	2,1	100	synchrotron	[44]
*N*‐acetyl‐l‐4‐hydroxyproline⋅H_2_O (HYPRO)	*P*2_1_2_1_2_1_	4,2	100	MoKα	[34]
			150	neutron	[34]

[a] The radiation used is also given. RT=room temperature. [b] The structure of l‐phenylalanine crystallizes as l‐phenylalanine‐l‐phenylalaninium formic acid.

Detailed analyses of all of the datasets including a topological analysis of the electron density distribution^46^ were previously published.^37^, ^39^, ^41^, ^44^ In terms of experimental charge‐density determination, these results remain valid and up to date. These data sets comprise an excellent test set for method development. Since diffraction data were never deposited, we supply them as supplementary information and use the opportunity to correct for three sources of systematic errors that were not taken into account in some of our earlier studies, namely, an empirical correction for thermal diffuse scattering using methods recently described,^47^ probably also partly correcting for the oblique incidence,^48^ and the non‐linearity of the first generation of charged coupled device (CCD) area detectors. Final data were, if required, converted into intensities and subsequently merged with xprep.

### Refinement with xd

In our current least‐squares aspherical‐atom refinements we decided not to adjust multipole populations to the experimental diffraction data. To ensure a consistent model, aspherical scattering factors of the invariom database^23^ were used instead. This procedure also avoids any possible ambiguity for molecules crystallizing in non‐centrosymmetric space groups.^49^ Refinements were carried out on *F* ^2^,^50^, ^51^ computing *R*_1_(*F*) for all reflections, rather than the 3 *σ* cutoff on *F* employed before. In these refinements the same invariom scattering factors were assigned for atoms in chemically analogous covalent bonding environments.^52^ The xd2006 software^53^ was employed, preceded by the preprocessor invariomtool 54 to generate xd input files. The program APD‐toolkit 35 enabled us to estimate H‐ADPs, relying on the same model compounds and computations of the invariom database as for generating the scattering factors. Radial functions of all atoms were taken from the SCM set.^55^ Charge transfer between water solvent and amino acid molecules was not allowed. Figures of merit remain comparable to results reported previously, despite not adjusting the multipole populations to the intensity data. The conclusion can be drawn that classical charge‐density studies are only worth the considerable effort for molecules with unconventional bonding environments or strong crystal‐field effects; we find free refinement especially relevant in cases where theory and experiment are expected to disagree.

### Hirshfeld atom refinement with tonto

Hirshfeld atom refinement was then performed on the asymmetric‐unit content starting from the coordinates and H‐ADPs after invariom refinement. Two sets of refinement were carried out: the first adjusted positions and ADPs of all atoms. In the second, only positions and non‐hydrogen ADPs were refined, keeping the TLS+INV estimated H‐ADPs fixed. Scattering factors used were derived from BLYP/cc‐pVTZ^56^ wavefunctions. To generate an approximate crystal field, atomic point charges and dipoles were placed surrounding the central molecule. A cluster radius of approximately 15 Å was employed. Point charges and dipoles were obtained from Hirshfeld partitioning.^15^

## Cluster QM/MM computations

ONIOM (“our own n‐layered integrated molecular orbital and molecular mechanics”) computations^57^ were carried out for each asymmetric unit content of the structures of the compounds given in Table 1. These computations provide accurate bond lengths to hydrogen atoms in their crystalline environments at moderate computational cost. B3LYP/cc‐pVTZ:UFF ONIOM computations took less than two days on a 12‐core 2.93 GHz Xeon workstation, so that the computational effort can be considered moderate (and is actually similar to HAR despite the different purpose). Input files for Gaussian 09^58^ were generated with the utility baerlauch,^59^ and require only fractional atomic coordinates, a distance cutoff radius, the universal force field (UFF) atom types^60^ to be assigned to the atoms in the asymmetric unit and the crystal space group. Only the asymmetric unit molecules (usually one zwitterionic amino acid and for the hydrates an additional water molecule) were included in the high layer (that is, the better level of theory) of the ONIOM calculation; for l‐phenylalanine‐l‐phenylalaninium formic acid, only one zwitterionic l‐phenylalanine molecule was chosen to be the high layer. Each high‐layer was then energy minimized at the DFT level of theory [functional/basis set: B3LYP/cc‐pVTZ], while all other molecules were part of the low layer as described by UFF. To ensure that convergence is successful, computations were initially performed with the basis set 6‐31G(d,p). Electrostatic embedding was included for the respective basis‐set choice for the surrounding molecules; the required RESP point charges^61^ were obtained from a preceding single‐point computation on the experimental input structure. Concerning cluster sizes, most clusters could be generated with the default distance cutoff of 3.75 Å. It applies to distances between atoms within the asymmetric unit to any atom surrounding them; all molecules with atoms within that distance were included. The cutoff‐distance usually led to the generation of clusters of 15 molecules (except for d,l‐glutamic acid monohydrate with 16 and d,l‐serine with 14 molecules). For d,l‐lysine hydrochloride (refcode: DLLYSC11), which was also studied earlier,^37^ convergence could only be reached using the Hartree–Fock method and a significantly larger cluster size of 30 molecules, which is why it was excluded from the study. l‐Threonine and l‐homoserine^62^ were previously investigated by a similar method;^59^ for l‐threonine the aim was to compute ADPs, for l‐homoserine to study the crystal field effect and compare bond lengths to hydrogen atoms.

### Statistical methods

Given a set of *N* values *V*={*V_i_* }, the mean value and its population standard deviation are defined by Equation (1):

(1) $$\left\langle V\right\rangle =\frac{1}{N}\overset{N}{\underset{i=1}{\sum }}V{}_{i}$$

The population standard deviation *σ*_pop_ or root mean‐square deviation (RMSD) gives an indication of the spread of the values around the mean (Equation (2)).

(2) $$\sigma {}_{\mathrm{pop}}\left(V\right)=\sqrt{\frac{1}{N}\left(\overset{N}{\underset{i=1}{\sum }}V{}_{i}{}^{2}\right)-\left\langle V\right\rangle {}^{2}}$$

The error in the mean is given by Equation (3):

(3) $$\sigma {}_{\mathrm{mean}}\left(V\right)=\frac{\sigma {}_{\mathrm{pop}}\left(V\right)}{\sqrt{N-1}}$$

Herein several pairs of bond lengths are compared. These are derived from neutron or X‐ray measurements as well as ONIOM computations, denoted {*N_i_* }, {*X_i_* }, and {*O_i_* }. We follow earlier work^14^ and use the statistical measures to describe similarities and differences. In the following comparisons the X‐ray or ONIOM value to be compared {*C_i_* } is subtracted from the neutron value when this is available, so that a positive value indicates that the X‐ray or ONIOM result is too short. When neutron values are not available, the quantum chemical ONIOM result is chosen as benchmark {*B_i_* } for the X‐ray results. Following values for the combined set *V* are reported with the following nomenclature:

The mean difference (MD), denoted ⟨*δP*⟩, is associated with the set *V*=Δ*P*, where *C_i_* can either be X‐ray {*X_i_* } or ONIOM {*O_i_* } results. The MD is also known as the signed difference. The MD can be positive or negative, meaning that on average the parameters derived from the X‐ray measurements or ONIOM computations are smaller or larger, respectively, than those derived from the neutron measurements.

The mean of the square of the weighted difference, weighted by the combined standard uncertainties from both measurements, is denoted ⟨[Δ*P*/csu(*P*)]^2^⟩. It is associated with the set *V*={[(*B_i_*−*C_i_* )/csu(*B_i_*,*C_i_* )]^2^}. The combined standard uncertainty (csu), which appears in this expression, is given by Equation (4):^63^

(4) $$\mathrm{csu}\left(B{}_{i},C{}_{i}\right)=\sqrt{\mathrm{su}\left(B{}_{i}\right){}^{2}+\mathrm{su}\left(C{}_{i}\right){}^{2}}$$

Combining these equations, the mean of the square of the weighted difference is given by Equation (5):

(5) $$\left\langle {\left[\frac{B{}_{i}-C{}_{i}}{\sqrt{\mathrm{su}\left(B{}_{i}\right){}^{2}+\mathrm{su}\left(C{}_{i}\right){}^{2}}}\right]}^{2}\right\rangle$$

For reasons of convention, we report the square root of this property and refer to it as the csu‐weighted root mean‐square difference (wRMSD). For ONIOM results the standard deviation was used as zero.

## Results

Aspherical atom refinement (HAR and INV alike) leads to improved figures of merit (Table 2), lower average Hirshfeld test results,^64^ and thus more physically meaningful displacement parameters. Such results have already been obtained in a number of earlier studies on organic compounds,^13^, ^14^, ^17^, ^65^, ^66^, ^67^ so that similar results for the compounds studied here will not be discussed in detail. Atomic displacement tensors are also not compared, since the measurement temperature and resolution of the experiments differ. Instead, we focus on bond lengths to hydrogen atoms and the best procedure to obtain these. We note in passing that *R*_all_(*F*) is very similar for both types of aspherical‐atom refinement.

**Table 2:** Selected dataset characteristics for the nine structures studied.^[a]^

Structure	Resolution (sin *θ*)/*λ* [Å^−1^]	*R*_all_(*F*) (INV)^[b]^ [%]	*R*_all_(*F*) (HAR)^[c]^ [%]
d,l‐asparagine⋅H_2_O	1.46	4.64	4.58
d,l‐glutamic Acid⋅H_2_O	1.30	9.43	9.35
l‐glutamine	1.08	2.16	2.17
l‐phenylalanine	1.17	3.46	–
d,l‐proline⋅H_2_O	1.12	5.05	5.10
d,l‐serine	1.54/1.18	8.70/2.69	–/3.23
l‐threonine	1.35	4.29	4.29
d,l‐valine	1.54	6.25	6.01
*N*‐acetyl‐l‐4‐hydroxyproline⋅H_2_O	1.13	6.16	5.82

[a] The weighting scheme was of 1/*σ* ^2^ in all cases. [b] INV=invariom. [c] HAR=Hirshfeld atom refinement with H‐ADPs estimated.

Hydrogen ADPs from HAR with cluster charges and dipoles can in principle include and describe hydrogen bonding and crystal‐field effects. In refinements performed prior to this work, positions and ADPs of all atoms have been chosen to be adjustable parameters. Including H‐ADPs as model parameters improves the accuracy of X−H bond lengths in comparison to the isotropic description of hydrogen atom displacements (the fit to the diffraction data usually improves as well).^18^ We would, however, advise not using refined H‐ADPs for HAR refinement for accurate structure determinations in general (the authors were reminded of earlier problems with ADPs of non‐hydrogen atoms^68^), since these refinements give non‐positive definite hydrogen ADPs in five out of the nine molecules studied. ORTEP plots for l‐asn, l‐gln, l‐phe, d,l‐pro⋅H_2_O, and l‐thr (Supporting Information) clearly show that they become oblate. (Technically it is impossible to visualize them when they are NPD, so the depiction is not strictly that of an NPD H‐ADP, but what the program platon prints.) The reason for the problems in simultaneous adjustment of positional and displacement parameters for hydrogen atoms is over‐parameterization. This occurs even for datasets that have been measured carefully, and to exceptionally high resolution. Free refinement can therefore not be considered a recommendable procedure for everyday use; as stated in the introduction the real issue is not only low‐order data quality and completeness, but the limited extent of hydrogen‐atom scattering (see Figure 1). Hence no additional information becomes available on hydrogen positions when including high‐order data in XRD, in contrast to neutron diffraction, where the scattering lengths are constant. Only X‐ray data collected at very low temperatures might permit H‐ADP refinement.

Concerning bond lengths to hydrogen atoms from single‐crystal XRD, we therefore recommend using TLS+INV‐estimated H‐ADPs to avoid over‐parameterization.

We next discuss resulting bond lengths to hydrogen atoms obtained from three methods: ONIOM computations, and neutron and X‐ray diffraction evaluated by aspherical scattering factors. All give results in good agreement. We omit results from HAR where H‐ADPs become non‐positive definite. In those cases we use TLS+INV estimates of H‐ADPs as fixed parameters (that is, constraints) for positional refinement only. Otherwise these parameters are also freely adjusted in HAR, and two comparative sets of values are provided.

Capelli et al.^14^ have already shown that the precision of current neutron and X‐ray data is nearly the same for hydrogen atom positions (and is much better for non‐hydrogen atoms in X‐ray data). It was also shown that X‐ray X−H distances agree better with neutron results when using DFT rather than the Hartree–Fock method, and that a triple‐*ζ* basis set is sufficiently extended. We therefore used the BLYP/cc‐pVTZ method/basis set combination for HAR.

We will next discuss the accuracy of X‐ray aspherical atom refinements and the best procedures to extract accurate X−H bond lengths in more detail. Assessing the accuracy of structural data from several sources requires taking into account the standard uncertainty of both sets of experimental of X−H bond lengths, X‐ray and neutron. For first‐principle calculations standard deviations are not available. Like in earlier work on HAR,^14^ the combined standard uncertainty [csu; Eq. (4)] is taken into account in computing a weighted root mean square difference [wRMSD; Eq. (5)] and its square root [Eq. (3)]. It is essential in the assessment on whether the findings are significant, and in judging on how well neutron and X‐ray results agree overall.

Most relevant will be to compare for each sample listed the mean averaged difference (MD) and its population standard deviation *σ*_pop_ only for the X−H bonds (with X=C,N,O). These values are provided in Table 3. In the Supporting Information, MD values and their *σ*_pop_ for all X−X bonds including hydrogen are also given. Table 3 shows MD results for the three sources of bond lengths: single crystal neutron, X‐ray, and ONIOM. As pointed out before, we subtract X‐ray from neutron results (in contrast to previous work^14^), so that the mean average differences (MD) are positive when X−H bond lengths are shorter from for example, X‐ray diffraction. Mean absolute differences are not discussed. In the Supporting Information all individual bond lengths from all available methods are provided for each compound, together with invariom names for the hydrogen atoms.

**Table 3:** Statistical analysis of X−H bond lengths of the five structures where neutron data are available.^[a]^

MD, *σ*_pop_(MD)					
Structure	*N*,*X*_INV_	*N*,*X*_HARfree_	*N*,*X*_HAR_TLS+INV_	*N*,*O*	No. of data
ASN	0.0151, 0.0268	–	−0.0070, 0.0103	−0.0019, 0.0116	10
GLN	0.0157, 0.0136	0.0060, 0.0138	0.0060, 0.0106	0.0040, 0.0066	10
SER	0.0134, 0.0141	0.0260, 0.0240	0.0194, 0.0178	0.0054, 0.0074	7
THR	0.0426, 0.0376	–	0.0324, 0.0445	0.0052, 0.0116	9
HYPRO	0.0554, 0.0274	0.0465, 0.0255	0.0442, 0.0248	0.0006, 0.0133	13

[a] Analysis is through the mean difference MD ⟨Δ*P*⟩ and its population standard deviation *σ*_pop_. These values are reported for data pairs (*N*,*X*_INV_), (*N*,*X*_HARfree_), (*N*,*X*_HAR_TLS+INV_), and (*N*,*O*) when available. The number of data in each case is also given.

For the data of highest quality, the agreement between neutrons and ONIOM is superior to that in between neutron and X‐ray data. Here the 100 K laboratory data for l‐glutamine (GLN) and the 19 K data of l‐threonine (THR) show most favorable agreements.

Only for l‐asparagine monohydrate can X−H distances from HAR_TLS+INV be seen that are longer than the neutron results. Most other X‐ray refinements still give X−H bond lengths slightly shorter than the neutron result, which usually leads to a positive MD value. HAR refinements are on average in slightly better agreement to neutron results than the INV values. With *σ*_mean_ of 0.009 for INV and 0.008 for HAR in the case of l‐asparagine monohydrate, these differences are significant. ONIOM results agree even better with the neutron data than the X‐ray results, but also underestimate the X−H bonds slightly when neutron diffraction is taken as benchmark. These results are again significant, since *σ*_mean_ [Eq. (3); for example of l‐asparagine monohydrate of 0.004] is smaller than the difference. Nevertheless, the overall agreement is quite good, especially taking into account that the neutron data were measured at room temperature. How well the wavelength was known accurately in the neutron experiments could have been a source of systematic error. From the results in Table 3 we can also conclude that even bond lengths involved in hydrogen bonding are well reproduced by the two‐layer ONIOM computation despite the approximations involved, for example, using electrostatic interactions between high and low layer rather than a whole wavefunction for all molecules in the cluster. We think that ONIOM results can therefore be used as an alternative to neutron diffraction for the purpose of obtaining benchmark X−H bond lengths. Full periodic computations have been shown to also provide such results,^7^ albeit at a higher computational cost. As a next step we therefore use the ONIOM results as benchmark for comparing the X−H bond lengths for those remaining four structures where neutron data are not available.

From Table 4 it can be seen that the X−H bond lengths from ONIOM are also slightly longer than those from invariom refinement, while those from HAR (where DFT and the BLYP functional was used) agree better, and show very good agreement; notably, agreement between ONIOM and X‐ray results for d,l‐proline monohydrate and d,l‐valine is even better than for the comparisons involving neutron data above in Table 3.

**Table 4:** Statistical analysis of X−H bond lengths for the four remaining structures.^[a]^

MD, *σ*_pop_(MD)				
Structure	*O*,*X*_INV_	*O*,*X*_HARfree_	*O*,*X*_HAR_TLS+INV_	No. of data
GLU	0.0233, 0.0172	0.0083, 0.0138	0.0071, 0.0102	11
PHE	0.0275, 0.0345	–	–	11
PRO	0.0098, 0.0117	–	0.0088, 0.0286	11
VAL	0.0061, 0.0082	−0.0034, 0.0119	−0.0049, 0.0118	11

[a] Analysis is analogous to Table 3. Values are reported for data pairs (*O*,*X*_INV_), (*O*,*X*_HARfree_), and (*O*,*X*_HAR_TLS+INV_), if available; that is, ONIOM results (*O*) serve as reference. The number of data in each case is also given. HAR convergence failed for cases where values are not provided.

Another even more important point emerges from Tables 3 and Table 4: using estimated H‐ADPs systematically improves the bond lengths to hydrogen atoms in comparison to simultaneous free refinement of positional and displacement parameters. This holds both for using the neutron data as well as the ONIOM computations as benchmark. These results are again significant, as the *σ*_mean_ of 0.003 (*O*,*X*_HAR_TLS+INV_) or 0.004 (*O*,*X*_HARfree_) shows for the example of d,l‐glutamine monohydrate in the comparison to ONIOM results. In the comparison to neutron results, the example of l‐glutamine with *σ*_mean_ values of 0.005 (for the comparison *O*,*X*_HARfree_) or 0.004 (for *O*,*X*_HAR_TLS+INV_) also supports this conclusion. Hence using estimated H‐ADPs can solve the problem of over‐parameterization and provides better results at the same time.

Table 5 contains (wRMSD)^1/2^ values for pairs of results for each particular structure to assess overall agreement. Martín et al.^69^ have shown that even for two data sets of the same structure, measured on different diffractometers, using different radiation or at different temperatures, values around or exceeding two are normal. The values allow the similarity of the results to be assessed in one number, with an expected value of one for statistical agreement. The best overall result is seen for d,l‐valine from ONIOM and invariom refinement. In this molecule the aliphatic side chain does not undergo classical hydrogen bonding, so that invarioms perform well.

**Table 5:** Values of the square root of the wRMSD, taking into account only X−H bonds (X=C,N,O).^[a]^

	$\sqrt{\mathrm{wRMSD}}$			
Structure	*B*,*X*_INV_	*B*,*X*_HARfree_	*B*,*X*_HAR_TLS+INV_	*N*,*O*
ASN	2.21	–	1.62	5.45
GLN	2.50	3.48	2.73	1.69
SER	2.24	4.20	3.29	8.92
THR	3.99	–	5.92	1.66
HYPRO	6.13	5.67	6.23	2.22
GLU	3.21	1.88	1.65	–
PHE	2.42	–	–	–
PRO	2.85	–	5.16	–
VAL	1.30	2.27	2.29	–

[a] Values are again provided for data pairs (*N*,*X*_INV_), (*N*,*X*_HARfree_), (*N*,*X*_HAR_TLS+INV_), and (*N*,*O*), for the structures where neutron data (*N*) are available. For the remaining structures benchmark values (*B*) are taken from ONIOM, leading to (*O*,*X*_INV_), (*O*,*X*_HARfree_), and (*O*,*X*_HAR_TLS+INV_) comparisons. The number of data points is as in Tables 3 and 4.

Table 6 finally compares X−H bond lengths for particular chemical environments, as defined by the invariom name. We chose the positively charged hydrogen atoms of the amino group (invariom H1n[1c1h1h]), methyl (H1c[1c1h1h]), methylene (H1c[1c1c1h]), and HC*R*_3_ hydrogen atoms (H1c[1c1c1c]) alongside aromatic (H@6c) as well as hydroxy and water hydrogen atoms (shelxl AFIX commands groups hydrogen atoms in a similar manner than the invariom name). Hydrogen atoms that are assigned the same invariom are evaluated together for all molecules studied. The number of values contributing to an entry is given in square brackets. Here the first question was whether similar behavior in O−H, N−H, and C−H bonds can be seen: we can indeed start to see that C−H distances are closer to each other than the N−H or O−H values, but that the root mean square deviation is rather high. N−H and O−H values are shorter than neutron or ONIOM values especially in INV refinements. Here hydrogen bonding would need to be taken into account for better results. Obviously individual classification beyond the invariom name does not seem to provide much additional information on X−H bond lengths yet, since the number of values for each bond is too small. Nevertheless, taken together the values for the particular invariom are closer than those for X−H bond lengths combined in Tables 3 and 4. That is an interesting result that confirms the philosophy behind the invariom approach and opens up the possibility of getting more accurate bond‐distance restraints from the structures in the CCDC when those are analyzed by invariom names. Such restraints would not be limited to X−H bonds, and would be useful for example, for refinement of low‐resolution data as frequently encountered in macromolecular structure refinement. Alternatively, target values for such restraints could also be taken from the geometry‐optimized model compounds of the invariom database, with a basis set‐dependence in the latter, and an IAM bias in the former. For the example of protein crystallography, invariom database bond‐distance restraints would be in many aspects preferable to the popular values currently used,^70^ for example, when the focus would be on property determination rather than on the best fit to the diffraction data. Results in Table 6 are just a beginning, and more work is certainly required.

**Table 6:** X−H bond lengths of individual invarioms characterized with the mean difference MD ⟨Δ*P*⟩ to and its population standard deviation *σ*_pop_.^[a]^

Invariom	MD, *σ*_pop_(MD)		
	*N*,*X*_INV_ {No. of data}	*N*,*X*_HARfree_ {No. of data}	*N*,*X*_HAR_TLS+INV_ {No. of data}
H1c[1o1c1c]	0.0430 (0.0090) {2}	0.0230 {1}	0.0200 (0.0080) {2}
H1n[1.5c1h]	0.0015 (0.0216) {4}	‐0.0020 (0.0160) {2}	0.0030 (0.0050) {4}
H1n[1c1h1h]	0.0332 (0.0315) {12}	0.0117 (0.0113) {6}	0.0160 (0.0378) {12}
H1c[1c1h1h]	0.0503 (0.0315) {6}	0.0380 (0.0014) {3}	0.0272 (0.0344) {6}
H1c[1c1c1h]	0.0147 (0.0219) {12}	0.0184 (0.0160) {10}	0.0107 (0.0191) {12}
H1c[1c1c1c]	0.0230 (0.0109) {5}	0.0197 (0.0150) {3}	0.0142 (0.0131) {5}
H1°[1c]	0.0587 (0.0354) {4}	0.0853 (0.0122) {3}	0.0625 (0.0244) {4}
H1°[1 h]	0.0417 (0.0356) {4}	0.0725 (0.0045) {2}	0.0337 (0.0371) {4}

	*O*,*X*_INV_ {No. of data}	*O*,*X*_HARfree_ {No. of data}	*O*,*X*_HAR_TLS+INV_ {No. of data}
H1n[1c1h1h]	0.0250 (0) {2}	– {0}	0.0420 (0.0100) {2}
H1c[1o1c1c]	– {0}	– {0}	– {0}
H1n[1.5c1h]	– {0}	– {0}	– {0}
H@6c	0.0142 (0.0150) {5}	– {0}	– {0}
H1n[1c1h1h]	0.0229 (0.0324) {9}	0.0028 (0.0135) {6}	0.0048 (0.0139) {6}
H1c[1c1h1h]	0.0041 (0.0054) {7}	−0.0003 (0.0080) {7}	−0.0051 (0.0071) {7}
H1c[1c1c1h]	0.0099 (0.0139) {12}	−0.0003 (0.0093) {4}	−0.0017 (0.0113) {10}
H1c[1c1c1c]	0.0225 (0.0278) {4}	−0.0075 (0.0085) {2}	−0.0163 (0.0140) {3}
H1°[1c]	0.0680 {1}	0.0120 {1}	−0.0010 {1}
H1°[1h]	0.0251 (0.0135) {4}	0.0300 (0.0130) {2}	0.0277 (0.0225) {4}

[a] Values are provided for data pairs *N*,*X*_INV_, *N*,*X*_HARfree_, and *N*,*X*_HAR_TLS+INV_ in the top part of the table and *O*,*X*_INV_, *O*,*X*_HARfree_, and *O*,*X*_HAR_TLS+INV_ in the bottom. The number of data contributing in each case is also given in square brackets. When only one value is available, *σ*_pop_ is not given.

## Discussion

Obtaining accurate bond lengths to hydrogen atoms in X‐ray diffraction with conventional as well as high‐resolution X‐ray data sets benefits from using H‐ADPs as constraints. The TLS+INV approach^35^ provides a generally applicable and convenient open‐source solution to estimate such H‐ADPs.

Qualitatively similar results can be obtained when comparing INV and HAR aspherical‐atom refinements (each with TLS+INV H‐ADPs). The computational cost is seconds for INV refinement, and hours or days for HAR; the latter provides superior results. The situation is not unlike in quantum chemical computations with basis sets of increasing sophistication, where more extended bases give better results accompanied with increasing computational effort. Here the Gaussian‐type cc‐pVTZ basis set does perform (and is expected to perform) better than the Hansen/Coppens multipole model. The latter can, however, partly compensate its smaller single‐*ζ* basis by the Slater functions it relies upon. Therefore HAR also yields systematically lower su values than INV refinement. An important advantage of multipole‐model electron density is that it can be tabulated using only 25 (+kappa) parameters, and does not involve any SCF computation at the refinement stage. Taking into account the considerable computational effort of HAR, which further increases in *Z*′>1 structures, its usefulness in small‐molecule structure refinement is limited at the current stage. Here implementing extremely localized molecular orbitals^71^ could speed up the procedures considerably. In contrast to HAR, least squares refinement using databases of scattering factors can also be used for macromolecular structures,^72^ since they never require a self‐consistent field (SCF) computation of the whole system studied. For small molecules, scattering‐factor databases can only approach the accuracy reached in HAR. Nevertheless, both routes permit a more detailed treatment of hydrogen‐atom scattering than the IAM. We are thus in favor of databases for routine applications, since for their use in refinement all that is required is identification of the chemical environment and assignment of a suitable scattering factor to the atom of interest from a lookup table.

An open question is the more routine treatment of coordination complexes. When heavy atoms dominate scattering, the frozen core approximation of the Hansen–Coppens multipole model leads to a decreasing suitability^73^ for improving a structural model compared to the IAM. Therefore the invariom approach initially focused on amino acids^52^ and then organic compounds in general.^23^ The invariom approach could in principle be combined with other scattering‐factor models, for example, the quantum‐chemical density model used in HAR or other alternatives like the virtual‐spherical‐charges model.^74^ Ideal would be an implementation alongside an aspherical scattering factor formalism in shelxl.^75^ More recently the ability to model coordination compounds^76^ was added on a case‐by‐case basis. Here the location of hydrogen atoms near heavier nuclei requires further study.

Since combining several specialist computer programs is currently required, another important matter that needs to be resolved is wide applicability and user‐friendliness of the procedures involved. Learning to use currently available programs probably takes too much time for users who are focusing on determining connectivity. We will therefore develop required functionality further to become one‐click procedures in combination with the shelxl 75 software in the next years. APD‐toolkit 76 is already interfaced to shelxl and stores pre‐calculated values for H‐ADP estimation for rapid use. However, H‐ADPs require aspherical scattering factors to be really useful, functionality currently lacking in shelxl, and this is also going to be addressed.

## Conclusion

Key points emerging from the comparison are:

1. X−H bond lengths from ONIOM computations agree favorably with neutron diffraction results. They can hence be used to provide reference solid‐state bond lengths to hydrogen atoms at moderate computational cost when neutron results are un‐available.
2. ONIOM computations are easier to perform than neutron diffraction measurements owing to the considerable effort involved in the latter. This holds especially when considering the limited number of neutron facilities and the difficulty to obtain suitable crystal samples. ONIOM computations only require an X‐ray structure (space group, fractional coordinates, normalized X‐H distances) as input.
3. X−H bond lengths from free refinement of H‐ADPs in HAR are systematically inferior to the ones from refinements incorporating estimated H‐ADPs from the (segmented‐body) TLS+INV approach.
4. Free refinement of H‐ADPs in HAR frequently leads to non‐positive definite displacements even with good datasets owing to the scattering properties of hydrogen. This can therefore not be recommended to become a general procedure in X‐ray diffraction.
5. X−H bond lengths from invariom refinement with H‐ADPs estimated from the (segmented‐body) TLS+INV approach are systematically shorter but agree well with HAR overall, although strong hydrogen bonding is not taken into account in the invariom‐model density. HAR leads to slightly lower bond‐length standard deviations and lower mean differences with respect to neutron diffraction.
6. INV results agree slightly less well to ONIOM results than HAR when ONIOM rather than neutron data are taken as benchmark.
7. However, HAR requires a considerably larger computational effort (hours or days) than INV (seconds or minutes, including the TLS+INV estimation of H‐ADPs). For obtaining results rapidly, tabulated scattering factors are to be preferred for refinement.

In summary, the all‐too‐well known shortcoming of X‐ray diffraction to provide short bond lengths to hydrogen atoms is history. It has been resolved by invariom refinement, and results can further be improved upon by Hirshfeld atom refinement. Simultaneous refinement of hydrogen ADPs and their positions frequently leads to over‐parameterization; it is thus unsuitable for everyday use. We recommend a constrained hydrogen‐atom treatment with predicted ADPs by the TLS+INV approach, resulting in best agreement for bond lengths to hydrogen atoms from quantum chemistry and X‐ray and neutron crystallography. This ensures that results are based on physically reasonable H‐ADPs. The invariom approach provides estimates of H‐ADPs as well as aspherical scattering factors from the same model compounds; computations provide both electron density and infrared frequencies that can be converted into H‐ADPs. Both invariom refinement and HAR can be applied to conventional data sets. Finally two‐layer QM/MM (B3LYP/cc‐pVTZ:UFF) ONIOM computations were shown to be a real alternative to neutron diffraction for providing benchmark values for bond lengths to hydrogen atoms at low computational cost.

## Supporting information

As a service to our authors and readers, this journal provides supporting information supplied by the authors. Such materials are peer reviewed and may be re‐organized for online delivery, but are not copy‐edited or typeset. Technical support issues arising from supporting information (other than missing files) should be addressed to the authors.

Supplementary
