# Supplementary material for: Accurate Bond Lengths to Hydrogen Atoms from Single‐Crystal X‐ray Diffraction by Including Estimated Hydrogen ADPs and Comparison to Neutron and QM/MM Benchmarks
Source: Chemistry. 2017 Mar 15;23(19):4605–14. doi: 10.1002/chem.201604705 (PMC5434951; doi:10.1002/chem.201604705)
Supplement: Supplementary file 1 — Supplementary [file CHEM-23-4605-s001.pdf]

CHEMISTRY
A European Journal

Supporting Information

Accurate Bond Lengths to Hydrogen Atoms from Single-Crystal X-ray Diffraction by Including Estimated Hydrogen ADPs and Comparison to Neutron and QM/MM Benchmarks

Birger Dittrich,*[a] Jens Lübben,[a] Stefan Mebs,[b] Armin Wagner,[c] Peter Luger,[b] and Ralf Flaig*[c]

chem_201604705_sm_miscellaneous_information.pdf

Supporting information for the paper: "Accurate bond distances to hydrogen atoms from single-crystal X-ray diffraction by including estimated hydrogen ADPs and comparison to neutron and QM/MM benchmarks"

December 8, 2016

Birger Dittrich[a]* (dittrich@hhu.de), Jens Lübben[a], Stefan Mebs[b], Armin Wagner[c], Peter Luger[b], Ralf Flaig[c]* (ralf.flaig@diamond.ac.uk)

[a]Heinrich-Heine Universität Düsseldorf, Institut für Anorganische Chemie und Strukturchemie, Material- und Strukturforschung, Gebäude: 26.42, Universitätsstraße 1, 40225 Düsseldorf, *Germany*
[b]Institut für Chemie und Biochemie - Anorganische Chemie der Freien Universität Berlin, Berlin, D-14195, *Germany*
[c]Diamond Light Source, Harwell Science and Innovation Campus, Didcot, OX11 0DE, *United Kingdom*

Structures and CCDC Refcodes

Merged diffraction data of the investigated structures are deposited alongside this publication. The respective CCDC refcodes [1] of the earlier CIF depositions that contain the relevant structural models for refinement are provided in the following Table 1.

Table 1: CCDC Refcodes for the structures investigated. Crystallographic information files for these can be downloaded to initiate refinement. The refcode for the neutron data of N-acetyl-L-4-Hydroxyproline·H_2O is POKKAD02. For L-Threonine the refinement results of the 19K data were not deposited in the CCDC. Here earlier structure of a 12K dataset [2] should provide input coordinates.

**Structure**	**CCDC Refcode**
D,L-Asparagine H_2O	ASPARM09
D,L-Glutamic Acid H_2O	CADVU
L-Glutamine	GLUTAM02
L-Phenylalanine	JOTKIM01
D,L-Proline H_2O	DLPROM02
D,L-Serine	DLSERN12
L-Threonine	LTHREO03
D,L-Valine	VALIDL03
N-acetyl-L-4-Hydroxyproline ·H_2O	POKKA09

Depictions of non-positive definite H-ADPs from Hirshfeld atom refinment for four amino acids

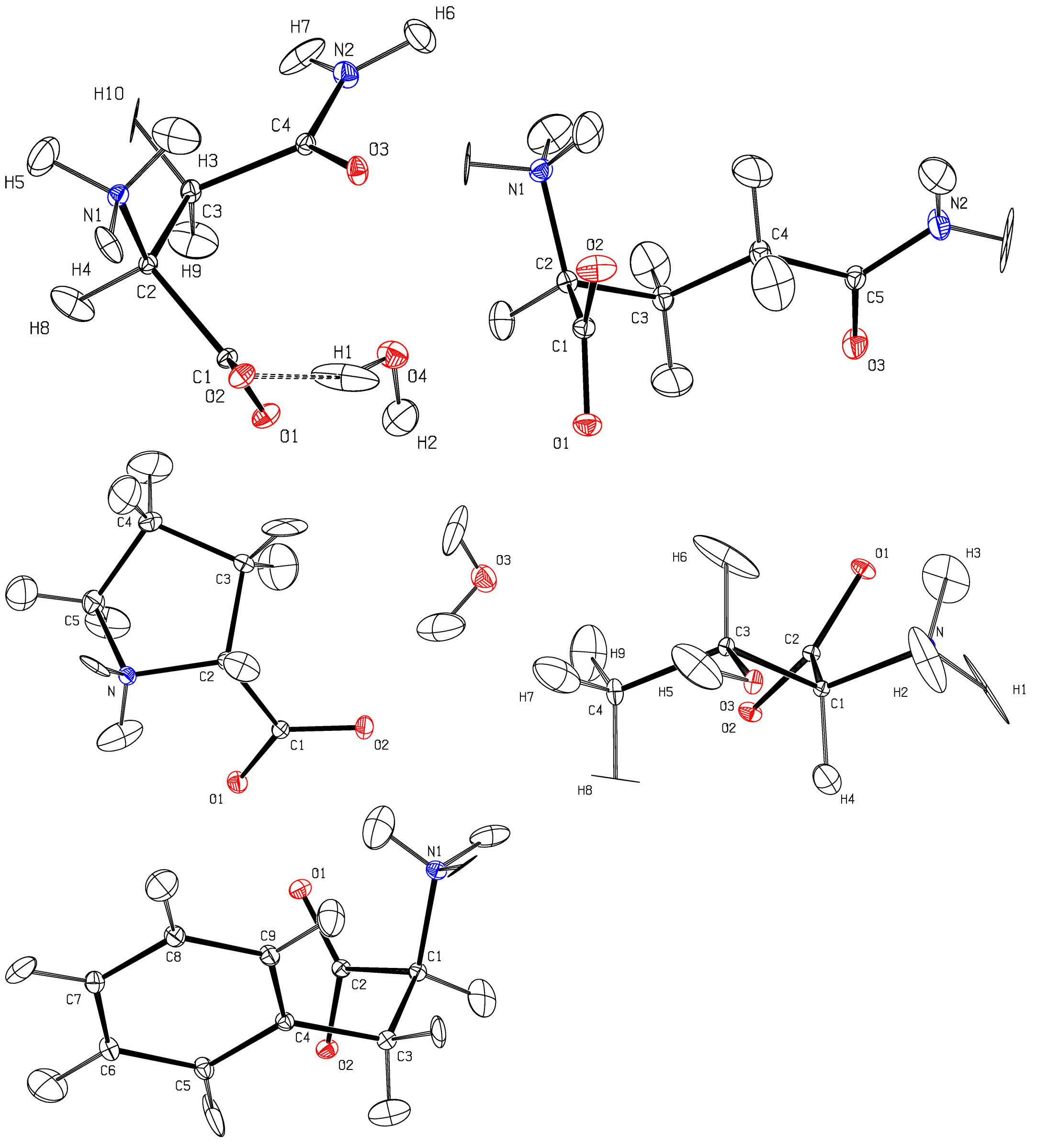

Figure 1: Depictions show experimental ORTEP-plots after Hirshfeld atom refinement. H-ADPs as well as H positions were adjusted freely to the X-ray data without using any further constraints or restraints. Figures generated with the program PLATON [3].

Statistical methods (also contained in the main article)

Given a set of N values $V = \{V_i\}$ the mean value and its population standard deviation are defined by:

$$\langle V \rangle = \frac{1}{N} \sum_{i=1}^{N} V_i \tag{1}$$

The population standard deviation σ_{pop} or root mean-square deviation (RMSD) gives an indication of the spread of the values around the mean.

$$\sigma_{pop}(V) = \sqrt{\frac{1}{N}\Big(\sum_{i=1}^{N} V_i^2\Big) - \langle V \rangle^2} \tag{2}$$

The error in the mean is given by:

$$\sigma_{mean}(V) = \frac{\sigma_{pop}(V)}{\sqrt{N-1}} \tag{3}$$

In this supplement several pairs of bond distances are compared, derived from neutron and X-ray measurements as well as ONIOM computations, denoted $\{N_i\}$, $\{X_i\}$ and $\{O_i\}$. We follow earlier work [4] and use the statistical measures to describe similarities and differences. In the following comparisons the X-ray or ONIOM value to be compared $\{C_i\}$ is subtracted from the neutron value when this is available, so that a positive value indicates that the X-ray or ONIOM result is too short. When neutron values are not available, the quantum chemical ONIOM result is chosen as benchmark $\{B_i\}$ for the X-ray results. Following values for the combined set V are reported with the following nomenclature:

(i) The mean difference (MD), denoted $\langle \Delta P \rangle$, is associated with the set $V = \Delta P = \{B_i - C_i\}$, where C_i can be either X-ray $\{X_i\}$ or ONIOM $\{O_i\}$ results. This quantity is also known as the signed difference. The MD can be positive or negative, meaning that on average the parameters derived from the X-ray measurements or ONIOM computations are smaller or larger, respectively, than those derived from the neutron measurements.

(ii) The mean of the square of the weighted difference – weighted by the combined standard uncertainties from both measurements – is denoted $\langle [\Delta P/csu(P)]^2 \rangle$. It is associated with the set $V = \{[(B_i - C_i)/csu(B_i, C_i)]^2\}$. The combined standard uncertainty (csu), which appears in this expression, is given by [5]

$$csu(B_i, C_i) = \sqrt{su(B_i)^2 + su(C_i)^2} \tag{4}$$

.

Combining these equations, the mean of the square of the weighted difference is

$$\Big\langle \Big\{ \frac{B_i - C_i}{\sqrt{su(B_i)^2 + su(C_i)^2}} \Big\}^2 \Big\rangle \tag{5}$$

For reasons of convention, we report the square root of this property and refer to it as the csu-weighted root mean-square difference (wRMSD). For ONIOM results the standard deviation was used as zero.

Detailed bond distances and discussion

In this supplement all bond distances in eight standard and one non-standard amino acids are listed in several tables. These are further analyzed by the MD using all atoms, whereas in the main paper these values were only reported for the bond distances involving hydrogen atoms. Subsequently bond distances of all molecules are discussed in detail case by case. In the main paper only the X—H distances are discussed and analysed statistically. Due to the small sizes of the molecules studied bond distances will be given in all cases.

We start the comparison using N-acetyl-L-4-hydroxyproline monohydrate. For this molecule neutron data at 150 K were available, which are used for comparison with high-resolution 100 K X-ray data. Three X-ray models were evaluated: INV refinement that relies on the Hansen/Coppens multipole model, HAR (free refinement of positions and H-ADPS) and HAR with refined hydrogen positions but fixed estimated TLS+INV H-ADPs (Table 2).

Table 2: Bond length (in Å) for N-acetyl-L-4-hydroxyproline monohydrate involving hydrogen atoms from quantum chemistry (ONIOM B3LYP/cc-pVTZ:UFF) for X-ray and neutron diffraction.

Bond	H-Invariom	Neutron (150 K)	ONIOM	INV (H-ADPs estd.)	HAR (H-ADPs free)	HAR (H-ADPs estd.)
O(1)—C(1)		1.211(3)	1.2144	1.2220(5)	1.2195(4)	1.2196(4)
O(2)—C(1)		1.308(3)	1.3291	1.3098(5)	1.3112(4)	1.3117(4)
O(2)—H(1)	H1o[1c]	1.011(5)	1.005	0.923(8)	0.909(7)	0.912(7)
O(3)—C(6)		1.241(4)	1.2365	1.2428(5)	1.2411(4)	1.2415(4)
O(4)—C(4)		1.421(3)	1.4243	1.4248(5)	1.4252(4)	1.4258(4)
O(4)—H(2)	H1o[1c]	1.003(6)	0.979	0.907(9)	0.930(9)	0.938(8)
O(5)—H(12)	H1o[1h]	0.971(8)	0.985	0.913(8)	0.894(8)	0.912(7)
O(5)—H(13)	H1o[1h]	0.952(5)	0.971	0.866(8)	0.884(7)	0.871(7)
N(1)—C(2)		1.460(2)	1.4618	1.4594(5)	1.4597(4)	1.4595(4)
N(1)—C(5)		1.468(3)	1.4735	1.4665(6)	1.4663(5)	1.4658(5)
N(1)—C(6)		1.339(3)	1.3493	1.3419(5)	1.3419(4)	1.3421(5)
C(1)—C(2)		1.522(3)	1.5227	1.5128(6)	1.5139(4)	1.5136(4)
C(2)—C(3)		1.541(4)	1.5438	1.5405(6)	1.5419(5)	1.5421(5)
C(2)—H(3)	H1c[1n1c1c]	1.099(4)	1.090	1.072(7)	1.073(5)	1.082(5)
C(3)—C(4)		1.529(3)	1.5318	1.5240(6)	1.5249(5)	1.5246(5)
C(3)—H(4)	H1c[1c1c1h]	1.085(5)	1.089	1.061(7)	1.071(5)	1.068(5)
C(3)—H(5)	H1c[1c1c1h]	1.102(6)	1.086	1.078(8)	1.078(6)	1.079(6)
C(4)—C(5)		1.532(3)	1.5332	1.5280(7)	1.5280(5)	1.5284(5)
C(4)—H(6)	H1c[1o1c1c]	1.093(6)	1.089	1.059(8)	1.070(6)	1.065(6)
C(5)—H(7)	H1c[1n1c1h]	1.105(6)	1.093	1.035(7)	1.048(7)	1.052(5)
C(5)—H(8)	H1c[1n1c1h]	1.091(6)	1.086	1.061(8)	1.064(6)	1.067(6)
C(6)—C(7)		1.500(3)	1.5032	1.4982(6)	1.4999(4)	1.4995(5)
C(7)—H(9)	H1c[1c1h1h]	1.089(7)	1.087	1.057(8)	1.050(7)	1.047(6)
C(7)—H(10)	H1c[1c1h1h]	1.072(9)	1.083	1.015(8)	1.033(7)	1.044(6)
C(7)—H(11)	H1c[1c1h1h]	1.068(7)	1.090	0.974(8)	1.032(8)	1.030(6)
MD(σ_{pop})		reference	-0.00174 (0.0108)	0.0289 (0.0341)	0.0241 (0.0298)	0.0229 (0.0286)

Bond distances from all five sources and approaches, invariom (with TLS+INV H-ADPs), HAR and HAR with TLS+INV H-ADPs (when necessary), neutron diffraction and ONIOM computations reasonably agree for N-acetyl-L-4-hydroxyproline monohydrate (Table 2), with the exception of a huge outlier for O(2)—C(1), where ONIOM overestimates the result.

The MD calculated for all pairs of bond distances using neutrons as reference shows that ONIOM results agree best with the neutron result. Concerning the X-ray results invariom refinement with TLS+INV H-ADPs shows a higher MD than HAR. Here estimated TLS+INV H-ADPs give the lowest MD, better than HAR with freely refined H-ADPs.

We next focus on the ONIOM results. For D,L-asparagine monohydrate, where neutron data collected at room temperature [6] are also available, the comparison likewise shows that neutron and ONIOM results are in best agreement for both molecules for all distances (including the X—H distances). The agreement can be less good for selected bond distances between heavier nuclei, and this will be discussed below for D,L-glu·H_2O. More remarkable are trends in the individual bond lengths involved in hydrogen bonding, which are well reproduced by the two-layer ONIOM computation despite the approximation of using electrostatic interactions between high and low layer rather than a whole wave function for all molecules in the cluster only. We conclude that ONIOM results can be used as an alternative to neutron diffraction in general, as shown using the examples of the genetically encoded amino acids in this work.

Table 3: Bond length (in Å) for D,L-asparagine monohydrate involving hydrogen atoms from quantum chemistry (ONIOM B3LYP/cc-pVTZ:UFF), neutron and X-ray diffraction.

Bond	H-Invariom	Neutron	ONIOM	INV (H-ADPs estd.)	HAR (H-ADPs estd.)
O(1)—C(1)		1.261(1)	1.2581	1.2649(4)	1.2653(3)
O(2)—C(1)		1.243(1)	1.2492	1.2458(3)	1.2459(3)
O(3)—C(4)		1.240(1)	1.2294	1.2421(3)	1.2418(3)
O(4)—H(1)	H1o[1h]	0.957(3)	0.965	0.923(15)	0.959(10)
O(4)—H(2)	H1o[1h]	0.962(3)	0.978	0.973(14)	0.965(9)
N(1)—C(2)		1.490(1)	1.5222	1.4912(4)	1.4915(3)
N(1)—H(3)	H1n[1c1h1h]	1.030(2)	1.056	0.978(13)	1.053(9)
N(1)—H(4)	H1n[1c1h1h]	1.040(2)	1.033	0.999(13)	1.048(8)
N(1)—H(5)	H1n[1c1h1h]	1.039(2)	1.027	0.994(14)	1.053(11)
N(2)—C(4)		1.332(1)	1.3462	1.3335(4)	1.3330(3)
N(2)—H(6)	H1n[1.5c1h]	1.026(2)	1.018	1.060(16)	1.026(11)
N(2)—H(7)	H1n[1.5c1h]	1.009(3)	1.016	1.004(16)	1.012(10)
C(1)—C(2)		1.538(1)	1.5639	1.5362(4)	1.5363(4)
C(2)—C(3)		1.522(1)	1.5203	1.5207(4)	1.5214(3)
C(2)—H(8)	H1c[1n1c1c]	1.091(2)	1.091	1.077(13)	1.087(7)
C(3)—C(4)		1.518(1)	1.5189	1.5180(4)	1.5178(4)
C(3)—H(9)	H1c[1c1c1h]	1.091(2)	1.088	1.098(13)	1.098(8)
C(3)—H(10)	H1c[1c1c1h]	1.087(2)	1.088	1.084(13)	1.110(6)
MD(σ_{pop})		reference	-0.00462(0.0131)	0.00792 (0.0216)	-0.00439 (0.00833)

Concerning the X-ray results for D,L-asparagine monohydrate (Table 3) values are only given for INV and HAR with TLS+INV estimated H-ADPs to avoid results based on non-positive definite H-ADPs (depicted in Figure 1). Therefore four rather than five sets of values are provided. HAR results perform better than the ONIOM results in this molecule. The situation that non positive definite H-ADPs are obtained after HAR is similar for L-phenylalanine L-phenylalaninium formic acid, D,L-proline monohydrate and L-threonine, where ORTEP plots [7] plots are provided in Figure 1[1].

[1]Due to problems with the program tonto s.u.'s given for HAR had in some cases be computed from the CIF file output and may not always take into account the full information from the covariance matrix.

Table 4: Bond length (in Å) for D,L-serine involving hydrogen atoms from quantum chemistry (ONIOM B3LYP/cc-pVTZ:UFF), neutron and from X-ray diffraction.

Bond	H-Invariom	Neutron	ONIOM	INV (H-ADPs estd.) 100 K data from [8])	HAR (H-ADPs free) data from [8])	HAR (H-ADPs estd.) data from [8]
O(1)—C(1)		1.248(1)	1.2481	1.2561(2)	1.2526(3)	1.2527(3)
O(2)—C(1)		1.257(1)	1.2546	1.2622(2)	1.2628(3)	1.2628(3)
O(3)—C(3)		1.414(1)	1.4191	1.4217(3)	1.4197(3)	1.4199(3)
O(3)—H(4)	H1o[1c]	0.981(1)	0.991	0.962(9)	0.900(9)	0.926(9)
N(1)—C(2)		1.487(1)	1.5151	1.4900(2)	1.4905(3)	1.4905(3)
N(1)—H(11)	H1n[1c1h1h]	1.037(1)	1.035	1.001(9)	1.018(9)	1.015(9)
N(1)—H(13)	H1n[1c1h1h]	1.045(1)	1.035	1.035(9)	1.041(9)	1.044(9)
N(1)—H(12)	H1n[1c1h1h]	1.041(1)	1.031	1.025(8)	1.030(10)	1.039(9)
C(1)—C(2)		1.531(1)	1.5567	1.5325(3)	1.5321(3)	1.5320(3)
C(2)—C(3)		1.518(1)	1.5178	1.5218(2)	1.5224(3)	1.5224(3)
C(2)—H(2)	H1c[1n1c1c]	1.101(1)	1.086	1.080(8)	1.067(6)	1.068(6)
C(3)—H(32)	H1c[1o1c1h]	1.095(1)	1.091	1.108(8)	1.078(9)	1.084(9)
C(3)—H(31)	H1c[1o1c1h]	1.095(2)	1.088	1.090(8)	1.079(9)	1.083(8)
MD(σ_{pop})		reference	-0.00142 (0.0126)	0.00498 (0.0139)	0.0121 (0.0232)	0.0194 (0.0178)

For D,L-serine (Table 4, X-ray data from [8]) room temperature neutron data were taken from [9]. The invariom with TLS+INV bond distances improve compared to 2005 as listed in [8], where for hydrogen at the time only nearest neighbor atoms were considered in invariom model compounds, and where only isotropic displacement parameters were refined for them. In this study next-nearest neighbor model compounds [10] and the TLS+INV H-ADPs were used for hydrogen; including estimated H-ADPs can be considered an important improvement since X—H bond distances get closer to neutron diffraction and ONIOM results.

Table 5: Bond length (in Å) for D,L-serine involving hydrogen atoms from quantum chemistry (ONIOM B3LYP/cc-pVTZ:UFF), neutron and from X-ray diffraction.

Bond	H-Invariom	Neutron	ONIOM	INV (H-ADPs estd.) 100K data from [11]
O(1)—C(1)		1.248(1)	1.2481	1.2523(3)
O(2)—C(1)		1.257(1)	1.2546	1.2630(3)
O(3)—C(3)		1.414(1)	1.4191	1.4198(3)
O(3)—H(4)	H1o[1c]	0.981(1)	0.991	0.972(10)
N(1)—C(2)		1.487(1)	1.5151	1.4909(3)
N(1)—H(11)	H1n[1c1h1h]	1.037(1)	1.035	1.018(10)
N(1)—H(13)	H1n[1c1h1h]	1.045(1)	1.035	1.047(10)
N(1)—H(12)	H1n[1c1h1h]	1.041(1)	1.031	1.018(9)
C(1)—C(2)		1.531(1)	1.5567	1.5325(3)
C(2)—C(3)		1.518(1)	1.5178	1.5227(3)
C(2)—H(2)	H1c[1n1c1c]	1.101(1)	1.086	1.075(8)
C(3)—H(32)	H1c[1o1c1h]	1.095(1)	1.091	1.108(9)
C(3)—H(31)	H1c[1o1c1h]	1.095(2)	1.088	1.083(9)
MD(σ_{pop})		reference	-0.00142 (0.0126)	0.00368 (0.0121)

When we use the 100 K synchrotron data to unusually high resolution from [11] rather than the 100 K dataset from the multi-temperature laboratory data from 2005 (Table 5), very similar results are obtained (only shown for invariom refinement since HAR failed). The higher resolution synchrotron data giving better agreement with neutron diffraction than the MoKα data for INV refinement. The MD value findings indicate that agreement with neutron diffraction is again best for ONIOM, followed by Invariom refinement (Flaig's as well as Dittrich's X-ray dataset) with TLS+INV H-ADPs. For HAR free refinement of H-ADP (Dittrich's data) and positional parameters gives better results than from HAR refinement with H-ADPs estimated by the TLS+INV approach when all bond distances are evaluated rather than just the X—H bond distances like in the main paper.

Table 6: Bond length (in Å) for L-glutamine involving hydrogen atoms from quantum chemistry (ONIOM B3LYP/cc-pVTZ:UFF), neutron and X-ray diffraction.

Bond	H-Invariom	Neutron	ONIOM	INV (H-ADPs estd.)	HAR (H-ADPs free)	HAR (H-ADPs estd.)
O(1)—C(1)		1.260(3)	1.2520	1.2672(3)	1.2680(3)	1.2683(3)
O(2)—C(1)		1.238(3)	1.2438	1.2442(3)	1.2437(3)	1.2434(3)
O(3)—C(5)		1.228(3)	1.2270	1.2420(3)	1.2414(3)	1.2412(3)
N(1)—C(2)		1.496(2)	1.5183	1.4965(3)	1.4956(3)	1.4949(3)
N(1)—H(1)	H1n[1c1h1h]	1.023(4)	1.024	0.991(8)	1.026(6)	1.018(6)
N(1)—H(2)	H1n[1c1h1h]	1.040(4)	1.037	1.023(8)	1.033(6)	1.034(6)
N(1)—H(3)	H1n[1c1h1h]	1.045(4)	1.040	1.041(8)	1.013(6)	1.022(6)
N(2)—C(5)		1.332(2)	1.3458	1.3377(3)	1.3370(3)	1.3364(3)
N(2)—H(9)	H1n[1.5c1h]	1.001(4)	1.004	0.990(8)	1.019(5)	0.996(6)
N(2)—H(10)	H1n[1.5c1h]	1.008(5)	1.000	0.984(8)	0.994(6)	0.998(6)
C(1)—C(2)		1.537(2)	1.5394	1.5422(3)	1.5431(2)	1.5434(3)
C(2)—C(3)		1.525(2)	1.5223	1.5331(3)	1.5342(2)	1.5346(3)
C(2)—H(4)	H1c[1n1c1c]	1.093(4)	1.084	1.057(7)	1.094(5)	1.101(5)
C(3)—C(4)		1.517(3)	1.5263	1.5246(3)	1.5230(3)	1.5224(3)
C(3)—H(5)	H1c[1c1c1h]	1.101(5)	1.084	1.090(7)	1.078(5)	1.077(5)
C(3)—H(6)	H1c[1c1c1h]	1.096(4)	1.087	1.068(6)	1.089(5)	1.090(5)
C(4)—C(5)		1.509(2)	1.5142	1.5152(3)	1.5172(3)	1.5179(3)
C(4)—H(7)	H1c[1c1c1h]	1.091(5)	1.092	1.100(7)	1.085(5)	1.092(5)
C(4)—H(8)	H1c[1c1c1h]	1.085(5)	1.091	1.082(6)	1.092(6)	1.095(5)
MD(σ_{pop})		reference	-0.000347 (0.0089)	0.0057 (0.0151)	0.0000632 (0.0121)	0.0000263(0.0121)

Room temperature neutron results [12] are again available for L-glutamine (Table 6), and here free refinement of H-ADPs was also possible in HAR, providing five sets of values for comparison. Here the MD for all X—X bond distances agrees most favorably with those by HAR with TLS+INV H-ADPs and free HA refinement, followed by those computed by ONIOM. It can be noted that for L-glutamine both X—H (main paper) as well as X—X bond distances (the value given in Table 6 from all sources agree very favorably overall.

Table 7: Bond length (in Å) for D,L-glutamic acid monohydrate involving hydrogen atoms from quantum chemistry (ONIOM B3LYP/cc-pVTZ:UFF) and X-ray diffraction.

Bond	H-Invariom	ONIOM	INV (H-ADPs estd.)	HAR (H-ADPs free)	HAR (H-ADPs estd.)
O(1)—C(2)		1.2569	1.2432(3)	1.2430(3)	1.2427(3)
O(2)—C(2)		1.2607	1.2704(3)	1.2704(3)	1.2705(3)
O(3)—C(5)		1.2140	1.2146(4)	1.2143(3)	1.2142(3)
O(4)—C(5)		1.3531	1.3320(3)	1.3322(3)	1.3325(3)
O(4)—H(9)	H1o[1c]	1.014	0.946(10)	1.002(10)	1.015(10)
O(5)—H(10)	H1o[1h]	0.974	0.941(8)	0.957(10)	0.960(8)
O(5)—H(11)	H1o[1h]	0.969	0.930(8)	0.926(9)	0.941(8)
N(1)—C(1)		1.5335	1.4893(3)	1.4890(3)	1.4891(3)
N(1)—H(1)	H1n[1c1h1h]	1.041	1.026(10)	1.027(8)	1.022(8)
N(1)—H(2)	H1n[1c1h1h]	1.049	1.040(10)	1.046(7)	1.044(7)
N(1)—H(3)	H1n[1c1h1h]	1.046	1.042(11)	1.044(8)	1.042(8)
C(1)—C(2)		1.5657	1.5303(3)	1.5306(3)	1.5307(3)
C(1)—C(3)		1.5373	1.5327(3)	1.5329(3)	1.5329(3)
C(1)—H(4)	H1c[1n1c1c]	1.093	1.074(9)	1.092(7)	1.091(7)
C(3)—C(4)		1.5355	1.5267(4)	1.5268(3)	1.5266(4)
C(3)—H(5)	H1c[1c1c1h]	1.091	1.072(9)	1.089(8)	1.091(7)
C(3)—H(6)	H1c[1c1c1h]	1.094	1.069(8)	1.080(6)	1.078(6)
C(4)—C(5)		1.5166	1.5048(3)	1.5050(3)	1.5048(3)
C(4)—H(7)	H1c[1c1c1h]	1.094	1.083(9)	1.104(7)	1.101(7)
C(4)—H(8)	H1c[1c1c1h]	1.093	1.079(8)	1.100(7)	1.095(7)
MD(σ_{pop})		reference	0.0193 (0.0173)	0.011 (0.0151)	0.0104 (0.0136)

For hydrogen-bonded D,L-glutamic acid monohydrate (Table 7) the trend in agreement in the absence of neutron data is the same than for most of the preceeding cases: using ONIOM results as reference for computing the MD, INV refinement agrees less well than HAR (free refinement), which is again less good than HAR refinement with estimated H-ADPs; neutron data really do not seem to be required to validate X—H bond as well as X—X distances. However, unlike in neutral N-acetyl-L-4-hydroxyproline the C_α—N bond distance is an outlier in the theoretical ONIOM computations. It disagrees considerably with the X-ray bond distances in this zwitterionic structure.

Table 8: Bond length (in Å) for zwitterionic L-phenylalanine in the solvate L-phenylalanine L-phenylalaninium formic acid from quantum chemistry (ONIOM B3LYP/cc-pVTZ:UFF) and X-ray diffraction.

Bond	H-Invariom	ONIOM	INV (H-ADPs estd.)
O(1A)—C(2A)		1.2568	1.2488(7)
O(2A)—C(2A)		1.2560	1.2667(6)
N(1A)—C(1A)		1.5247	1.4923(6)
N(1A)—H(11A)	H1n[1c1h1h]	1.048	0.95(2)
N(1A)—H(12A)	H1n[1c1h1h]	1.083	1.026(19)
N(1A)—H(13A)	H1n[1c1h1h]	1.030	1.043(18)
C(1A)—C(2A)		1.5560	1.5327(8)
C(1A)—C(3A)		1.5348	1.5390(7)
C(1A)—H(1A)	H1c[1n1c1c]	1.087	1.018(16)
C(3A)—C(4A)		1.5058	1.5114(8)
C(3A)—H(31A)	H1c[@6c1c1h]	1.085	1.049(16)
C(3A)—H(32A)	H1c[@6c1c1h]	1.090	1.105(18)
C(4A)—C(5A)		1.3970	1.4024(8)
C(4A)—C(9A)		1.3919	1.3976(7)
C(5A)—C(6A)		1.3896	1.3960(8)
C(5A)—H(5A)	H@6c	1.080	1.060(17)
C(6A)—C(7A)		1.3888	1.3948(8)
C(6A)—H(6A)	H@6c	1.083	1.062(19)
C(7A)—C(8A)		1.3861	1.3938(9)
C(7A)—H(7A)	H@6c	1.075	1.090(19)
C(8A)—C(9A)		1.3860	1.3960(8)
C(8A)—H(8A)	H@6c	1.078	1.061(18)
C(9A)—H(9A)	H@6c	1.079	1.051(19)
MD(σ_{pop})		reference	0.0133 (0.0291)

Like for D,L-glutamic acid monohydrate neutron data for the structure of L-phenylalanine L-phenylalaninium formic acid are unavailable and the C$_\alpha$—N bond distance is an outlier in the theoretical ONIOM computation (Table 8). The explanation that can be provided is the influence of the crystal field [13] that is only partly taken into account by point charges. The crystal field (including hydrogen bonding) causes oxygen atoms to polarize towards the positive carbon atom, while the H atoms polarize away from the negative N atom; polarization of the H-atoms thus leads to a weakening of the C_α—N bond and its elongated bond distance in the ONIOM computation. Similar polarizations have been visualized for L-homoserine, using different levels of model sophistication, starting from point charges, then improving the description with point charges and dipoles surrounding a molecule, and finally from full periodic DFT calculations [14]. Therefore only a MO description of the low-layer atoms in ONIOM or full periodic computations can give the correct bond distances from theory, at considerably higher computational effort. Because we are mainly interested in the X—H bond distances we consider the ONIOM B3LYP/cc-pVTZ:UFF levels of theory entirely appropriate here. HAR failed for L-phenylalanine L-phenylalaninium formic acid both for free refinement giving non-positive definite H-ADPs as well as for H-ADP-constrained refinement, where no minimum was found; X—H distances from INV refinement agree reasonably well with ONIOM results.

Table 10: Bond length (in Å) for L-threonine involving hydrogen atoms from quantum chemistry (ONIOM B3LYP/cc-pVTZ:UFF) and X-ray diffraction.

Bond	H-Invariom	Neutron	ONIOM	INV (H-ADPs estd.)	HAR (H-ADPs estd.)
O(1)—C(2)		1.237(4)	1.2524	1.2500(3)	1.2501(3)
O(2)—C(2)		1.258(4)	1.2526	1.2565(3)	1.2561(3)
O(3)—C(3)		1.416(4)	1.4266	1.4245(3)	1.4257(2)
O(3)—H(5)	H1o[1c]	0.983(6)	0.984	0.941(12)	0.952(11)
N(1)—C(1)		1.490(3)	1.5157	1.4895(3)	1.4895(3)
N(1)—H(1)	H1n[1c1h1h]	1.034(6)	1.026	0.957(12)	0.984(7)
N(1)—H(2)	H1n[1c1h1h]	1.024(6)	1.026	1.036(12)	1.022(7)
N(1)—H(3)	H1n[1c1h1h]	1.037(6)	1.049	0.935(13)	0.911(6)
C(1)—C(2)		1.539(3)	1.5492	1.5357(3)	1.5361(3)
C(1)—C(3)		1.538(3)	1.5358	1.5329(3)	1.5325(3)
C(1)—H(4)	H1c[1n1c1c]	1.096(6)	1.081	1.093(11)	1.080(7)
C(3)—C(4)		1.515(5)	1.5139	1.5201(4)	1.5205(3)
C(3)—H(6)	H1c[1o1c1c]	1.096(6)	1.093	1.055(12)	1.095(6)
C(4)—H(7)	H1c[1c1h1h]	1.097(9)	1.087	1.048(11)	1.069(6)
C(4)—H(8)	H1c[1c1h1h]	1.113(10)	1.088	1.038(12)	1.043(6)
C(4)—H(9)	H1c[1c1h1h]	1.075(10)	1.085	1.080(12)	1.118(7)
MD(σ_{pop})		reference	-0.000388 (0.0128)	0.0229 (0.0361)	0.0172 (0.0378)

Table 9: Bond length (in Å) for D,L-proline monohydrate involving hydrogen atoms from quantum chemistry (ONIOM B3LYP/cc-pVTZ:UFF) and X-ray diffraction.

Bond	H-Invariom	ONIOM	INV (H-ADPs estd.)	HAR (H-ADPs estd.)
O(1)—C(1)		1.2632	1.2667(4)	1.2661(4)
O(2)—C(1)		1.2400	1.2523(4)	1.2515(4)
O(3)—H(10)	H1o[1h]	0.965	0.9617(3)	0.960(8)
O(3)—H(11)	H1o[1h]	0.987	0.9618(3)	0.923(6)
N(1)—C(2)		1.5204	1.5035(4)	1.5036(5)
N(1)—C(5)		1.5023	1.5011(4)	1.5012(5)
N(1)—H(1)	H1n[1c1c1h]	1.033	1.008(11)	0.981(6)
N(1)—H(2)	H1n[1c1c1h]	1.038	1.013(10)	1.006(5)
C(1)—C(2)		1.5368	1.5286(4)	1.5292(4)
C(2)—C(3)		1.5464	1.5475(5)	1.5466(6)
C(2)—H(3)	H1c[1n1c1c]	1.079	1.079(10)	1.111(6)
C(3)—C(4)		1.5299	1.5344(5)	1.5350(5)
C(3)—H(8)	H1c[1c1c1h]	1.087	1.082(11)	1.070(5)
C(3)—H(9)	H1c[1c1c1h]	1.085	1.079(11)	1.082(6)
C(4)—C(5)		1.5150	1.5243(5)	1.5246(5)
C(4)—H(6)	H1c[1c1c1h]	1.082	1.092(11)	1.104(6)
C(4)—H(7)	H1c[1c1c1h]	1.087	1.088(10)	1.100(5)
C(5)—H(4)	H1c[1n1c1h]	1.086	1.065(10)	1.088(5)
C(5)—H(5)	H1c[1n1c1h]	1.081	1.073(10)	1.088(5)
MD(σ_{pop})		reference	0.00543 (0.0118)	0.00491 (0.023)

Since there are no neutron results for D,L-proline monohydrate (Table 9) the reference to compare the X-ray data with has to be the result of the ONIOM computation. Invariom refinement shows a slightly less good agreement than HAR in terms of the MD when using all X—H and X—X bond distances. Optimized ONIOM bond distances come from a triple zeta basis set, as does HAR for the crystallographic refinement. I this regard the multipole model performs well despite the single Slater function per multipole — results are alost as good.

For L-threonine a room temperature neutron structure [15] provides reference bond distances (Table 10). Again the agreement between neutron diffraction and ONIOM computations is clearly most favorable (apart from the C_α—N bond), supporting the conclusion of the main paper that the latter can be used to provide comparative results for the other structures. The next-best agreement is HAR and then invariom refinement, both with with estimated TLS+INV H-ADPs.

Table 11: Bond length (in Å) for D,L-valine involving hydrogen atoms from quantum chemistry (ONIOM B3LYP/cc-pVTZ:UFF) and for X-ray diffraction.

Bond	H-Invariom	ONIOM	INV (H-ADPs estd.)	HAR (H-ADPs free)	HAR (H-ADPs estd.)
O(1)—C(1)		1.2463	1.25911(18)	1.2590(2)	1.2590(2)
O(2)—C(1)		1.2628	1.25759(19)	1.2577(2)	1.2576(2)
N(1)—C(2)		1.5190	1.49222(19)	1.4923(2)	1.4924(2)
N(1)—H(1)	H1n[1c1h1h]	1.028	1.026(9)	1.029(6)	1.031(7)
N(1)—H(2)	H1n[1c1h1h]	1.037	1.031(7)	1.059(5)	1.056(6)
N(1)—H(3)	H1n[1c1h1h]	1.049	1.021(8)	1.028(6)	1.026(6)
C(1)—C(2)		1.5544	1.53975(17)	1.5401(2)	1.5400(2)
C(2)—C(3)		1.5465	1.5456(2)	1.5454(2)	1.5454(2)
C(2)—H(4)	H1c[1n1c1c]	1.086	1.084(7)	1.103(5)	1.105(5)
C(3)—C(4)		1.5283	1.5307(2)	1.5308(2)	1.5308(2)
C(3)—C(5)		1.5260	1.5268(3)	1.5271(3)	1.5270(3)
C(3)—H(5)	H1c[1c1c1c]	1.095	1.085(7)	1.102(5)	1.099(5)
C(4)—H(6)	H1c[1c1h1h]	1.085	1.075(8)	1.089(6)	1.097(6)
C(4)—H(7)	H1c[1c1h1h]	1.089	1.083(8)	1.078(7)	1.085(7)
C(4)—H(8)	H1c[1c1h1h]	1.090	1.089(7)	1.104(5)	1.104(5)
C(5)—H(9)	H1c[1c1h1h]	1.082	1.088(8)	1.088(8)	1.091(5)
C(5)—H(10)	H1c[1c1h1h]	1.089	1.082(9)	1.082(7)	1.083(7)
C(5)—H(11)	H1c[1c1h1h]	1.086	1.085(8)	1.092(7)	1.093(6)
MD(σ_{pop})		reference	0.00547 (0.00984)	-0.000339 (0.0125)	-0.00127 (0.0127)

For D,L-valine, the last molecule studied, neutron results are missing and the ONIOM computation again provides the reference values. Free refinement for H-ADPs worked well this time, so that four sets of values can be compared. The free HAR refinement is almost spot on the QM/MM result. HAR with TLS+INV H-APDs and INV refinemnt perform almost equally well (Table 11), HAR bond distances to hydrogen atoms seem to be slightly overestimate compared to the ONIOM result, which agrees better with the invariom distances. When all bond distances are included HAR is still slightly better.

References

[1] C. R. Groom, I. J. Bruno, M. P. Lightfoot, S. Ward, *Acta Cryst. B* **2016**, *72*, 171–179.

[2] J. Janczak, P. Luger, D. Zobel, *Acta Cryst. C* **1997**, *53*, 1901–1904.

[3] A. L. Spek, *Acta Cryst. D* **2009**, *65*, 148–155.

[4] S. C. Capelli, H. B. Bürgi, B. Dittrich, S. Grabowsky, D. Jayatilaka, *IUCrJ.* **2014**, *1*, 361–379.

[5] D. Schwarzenbach, S. C. Abrahams, H. D. Flack, E. Prince, A. J. C. Wilson, *Acta Cryst. A* **1995**, *51*, 565–569.

[6] J. J. Verbist, M. S. Lehmann, T. F. Koetzle, W. C. Hamilton, *Acta Cryst. B* **1972**, *28*, 3006–3013.

[7] M. N. Burnett, C. K. Johnson, ORTEP-III, Oak Ridge Thermal Ellipsoid Plot Program for Crystal Structure Illustrations, tech. rep., Oak Ridge National Laboratory Report ORNL-6895, Oak Ridge, Tennessee, **1996**.

[8] B. Dittrich, C. B. Hübschle, M. Messerschmidt, R. Kalinowski, D. Girnt, P. Luger, *Acta Cryst. A* **2005**, *61*, 314–320.

[9] M. N. Frey, M. S. Lehmann, T. F. Koetzle, W. C. Hamilton, *Acta Cryst. B* **1973**, *29*, 876–884.

[10] B. Dittrich, C. B. Hübschle, K. Pröpper, F. Dietrich, T. Stolper, J. J. Holstein, *Acta Cryst. B* **2013**, *69*, 91–104.

[11] R. Flaig, T. Koritsánszky, J. Janczak, H.-G. Krane, W. Morgenroth, P. Luger, *Angew. Chem. Int. Ed.* **1999**, *38*, 1397–1400.

[12] T. F. Koetzle, M. N. Frey, M. S. Lehmann, W. C. Hamilton, *Acta Cryst. B* **1973**, *29*, 2571–2575.

[13] C. Gatti, V. R. Saunders, C. Roetti, *J. Chem. Phys.* **1994**, *101*, 10686–10696.

[14] B. Dittrich, E. Sze, J. J. Holstein, C. B. Hübschle, D. Jayatilaka, *Acta Cryst. A* **2012**, *68*, 435–442.

[15] M. Ramanadham, S. K. Sikka, R. Chidambaram, *Pranama* **1973**, *1*, 247–259.
